# Non-invasive faecal sampling reveals spatial organization and improves measures of genetic diversity for the conservation assessment of territorial species: Caucasian lynx as a case species

**DOI:** 10.1371/journal.pone.0216549

**Published:** 2019-05-10

**Authors:** Deniz Mengüllüoğlu, Jörns Fickel, Heribert Hofer, Daniel W. Förster

**Affiliations:** 1 Leibniz Institute for Zoo and Wildlife Research (IZW), Berlin, Germany; 2 Department of Biology, Chemistry, Pharmacy, Freie Universität Berlin, Berlin, Germany; 3 Institute for Biochemistry and Biology, University of Potsdam, Potsdam-Golm, Germany; 4 Department of Veterinary Medicine, Freie Universität Berlin, Berlin, Germany; University of Goettingen, GERMANY

## Abstract

The Caucasian lynx, *Lynx lynx dinniki*, has one of the southernmost distributions in the Eurasian lynx range, covering Anatolian Turkey, the Caucasus and Iran. Little is known about the biology and the genetic status of this subspecies. To collect baseline genetic, ecological and behavioural data and benefit future conservation of *L*. *l*. *dinniki*, we monitored 11 lynx territories (396 km^2^) in northwestern Anatolia. We assessed genetic diversity of this population by non-invasively collecting 171 faecal samples and trapped and sampled 12 lynx individuals using box traps. We observed high allelic variation at 11 nuclear microsatellite markers, and found no signs of inbreeding despite the potential isolation of this population. We obtained similar numbers of distinct genotypes from the two sampling sources. Our results indicated that first order female relatives occupy neighbouring territories (female philopatry) and that territorial male lynx were highly unrelated to each other and to female territorial lynx, suggesting long distance male dispersal. Particular male and female resident territorial lynx and their offspring (kittens and subadults) were more likely to be trapped than resident floaters or dispersing (unrelated) lynx. Conversely, we obtained more data for unrelated lynx and higher numbers of territorials using non-invasive sampling (faeces). When invasive and non-invasive samples were analysed separately, the spatial organisation of lynx (in terms of female philopatry and females and males occupying permanent ranges) affected measures of genetic diversity in such a way that estimates of genetic diversity were reduced if only invasive samples were considered. It appears that, at small spatial scales, invasive sampling using box traps may underestimate the genetic diversity in carnivores with permanent ranges and philopatry such as the Eurasian lynx. As non-invasive sampling can also provide additional data on diet and spatial organisation, we advocate the use of such samples for conservation genetic studies of vulnerable, endangered or data deficient territorial species.

## Introduction

Conservation of wildlife species often requires highly demanding practices such as habitat preservation and restoration, animal protection, animal relocation, captive breeding and reintroductions [1]. For data deficient animal populations it is difficult to devise efficient conservation measures because there is insufficient information on their ecological, demographic and genetic status [1]. Non-invasive sampling strategies such as collecting faecal samples can provide crucial information about diet, allostatic load, reproduction, genetic diversity, and the dynamics in animal populations [2–4]. Once samples have been collected, genetic markers such as mtDNA and microsatellites can be used to assess genetic variability [5], estimate levels of inbreeding and relatedness [6, 7] and quantify total and effective population sizes [8]. Data acquired from such conservation genetic studies provide important information for efficient conservation actions [9, 10].

Among lynx species, the Eurasian lynx *Lynx lynx* has the widest geographic distribution. Populations occur in a wide variety of habitats, ranging in the Palearctic region from Scandinavia and central Europe to far eastern Russia, and can also be found south of the 20^th^ degree of latitude (e.g., in southwest Asia and Tibet; [11]). Whereas mtDNA diversity has been characterized for some populations of this species [12], nuclear genetic data are only available for European populations of the Eurasian lynx, several of which are considered ‘endangered’ or ‘vulnerable’ [11, 13, 14].

The two subspecies of *Lynx lynx* in Asia, *L*. *l*. *dinniki* and *L*. *l*. *isabellinus*, are still little known in terms of their ecological requirements, spatial and genetic population structure and their genetic diversity. The Caucasian lynx *L*. *l*. *dinniki* (Satunin 1915) has one of the southernmost distributions of Eurasian lynx [11], stretching from the Anatolian side of Turkey to the Caucasus and Iran. Compared with their north and central European conspecifics, Caucasian lynx display some behavioural and morphological differences. They are lagomorph specialists (similar to the Iberian lynx *Lynx pardinus*), have a smaller body size [15] and smaller home range sizes, and thus occur in suitable habitats at higher population densities [16] than European subspecies. They live in dry open, rocky and coniferous habitats and scrape mark [15, 17], a marking behaviour that in the genus *Lynx* is otherwise only observed in bobcats (*Lynx rufus* [18]). Highway collisions, habitat fragmentation and poaching are the main factors threatening the Caucasian lynx across its range [19].

Previously, two phylogeographic studies included Caucasian lynx among sampled subspecies of the Eurasian lynx [12, 20]. Both reported high mtDNA haplotype diversity and both suggested the presence of a glacial refugium for Eurasian lynx in this region. They did neither assess genetic variability at nuclear loci, nor did they assess the potential effect of recent anthropogenic activities and environmental changes on this variability. Such information is valuable to plan and carry out efficient lynx conservation measures [14].

**Fig 1:**
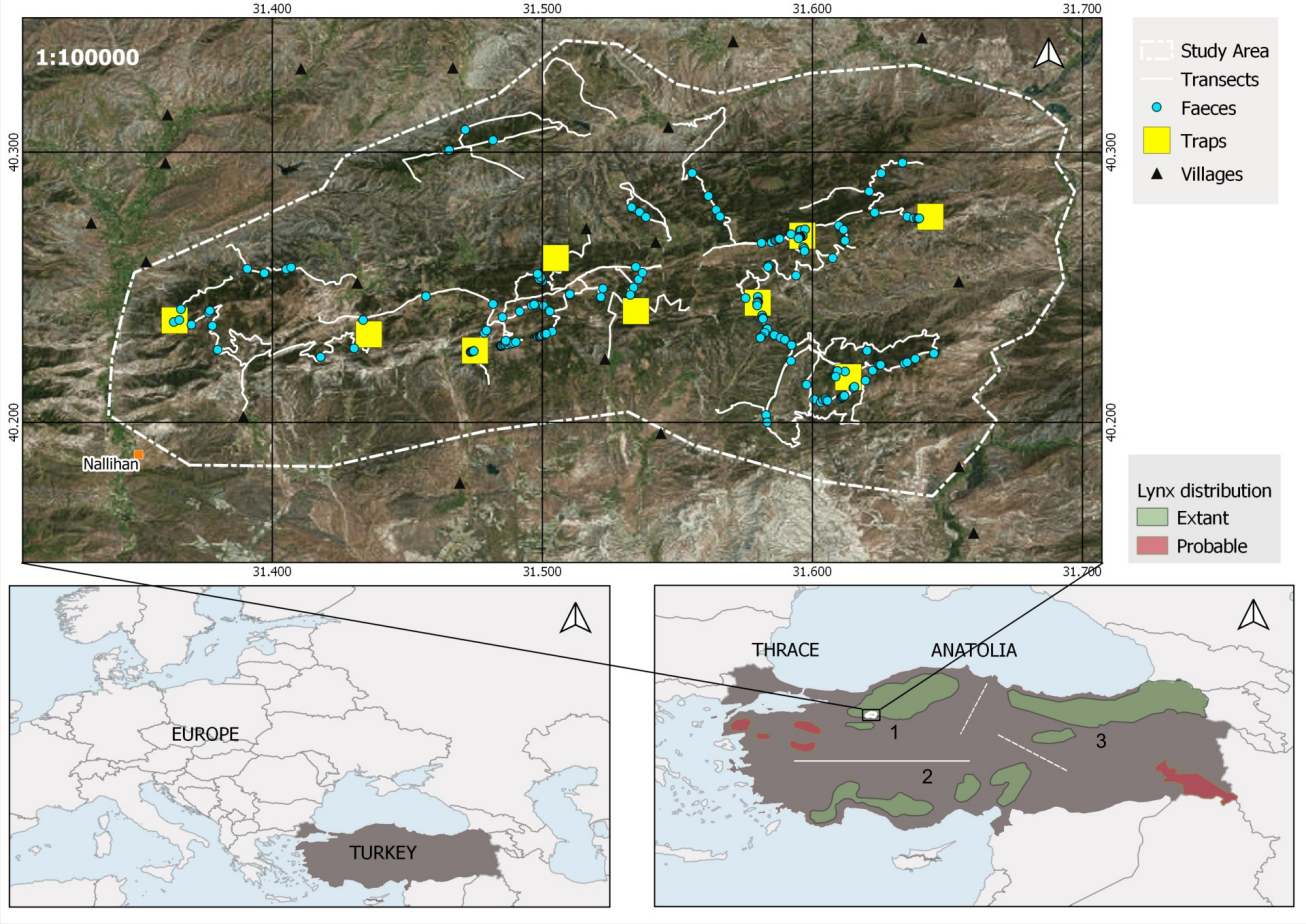
Location of the study area in north-west Anatolia and distribution of the three big extant lynx populations. 1: Northwestern, 2: southern, 3: northeastern lynx populations [11]. The continuous line indicates complete and dashed lines indicate potential isolation.

Three large Caucasian lynx populations occur in Turkey [11]. The northwestern Anatolian lynx population is isolated from the southern and northeastern populations by a series of natural and human constructed barriers (Fig 1). The inner Anatolian plateau with its agricultural landscape separates the northwestern lynx population from the southern population (Fig 1, continuous line), and a series of big dams (e.g. Seydim dam, Gökcedogan dam, Altinkaya dam) and human settlements separate it from the northeastern population (Fig 1, dashed line between 1 and 3). The southern population is isolated from the northeastern population by a series of rivers and dams situated in the deep valleys of the Anatolian diagonal mountain series (Fig 1, dashed line between 2 and 3). It is neither known whether there is gene flow between these populations, nor whether they are isolated and at risk from becoming genetically impoverished.

Non-invasive sampling enables researchers to collect samples in the field without disturbing the animals or putting them at health risk, and potentially represents a means to obtain genetic material from many individuals. For example, it may be feasible to collect faecal samples from a large area at a reasonable cost and effort. Genotyping success of non-invasively collected samples depends on several factors such as duration of exposure of the faeces to sun and humidity, as they affect the speed of DNA degradation, presence of PCR inhibitors [21], the amount of DNA in the sample originating from the study species [22], and the length of the DNA fragment (allele) to be amplified. Some of these factors are difficult to control for, such as the amount of inhibitors or the exposure to environmental conditions and aging of samples before collection, unless defecation is observed. Other factors such as collection procedure, storage and handling of the samples [23] are under the control of the investigator.

The collection of invasive samples can also be challenging. Its success depends on the population density of the study species and the trapping methodology used. The study design needs to take animal welfare into account and may be costly in terms of time and the resources required [24, 25]. Moreover, trapping success may depend on field experience with the study species, prior monitoring of the population to localise good trapping sites and the behavioural response of individuals to traps. In the case of many species with permanent ranges such as lynx, invasive sampling using boxes or cage trapping systems may require so much effort that inevitably the number of trapping locations will be locally restricted and confined to a small number of ranges or territories.

Some authors [26] have stressed the importance of a proper sampling scheme for the assessment of the genetic diversity in populations of philopatric animal species. They concluded that sampling at small spatial scales (“clumped sampling”) can produce results of apparently low genetic diversity and high relatedness among individuals. Considering that territoriality and female philopatry are common in many large carnivores, including the Eurasian lynx [27, 28], it is possible that genetic diversity measured at small spatial scales would be affected by spatial organization. However, populations of lynx (or other species) do not consist of territorial individuals only. Male lynx disperse long distances [27, 29] and females will also disperse if all the areas adjacent to the natal range are occupied [27]. In addition, there can be animals with large home ranges that are ‘resident’ and waiting to take over a local territory, often termed ‘floaters’–a recent example amongst felids is that of the cheetah (*Acinonyx jubatus*) where both territorial animals and floaters were identified as constituting the population of residents [30].

To our knowledge, no study has followed up on this idea and actually compared how sampling source (invasive vs. non-invasive) might influence the assessment of genetic diversity measures in philopatric animal populations where adults occupy permanent ranges and thus are not randomly distributed. Since stationary trapping systems such as box traps are more likely to capture territorial residents as these are habituated to the presence of traps, we hypothesise that non-invasively collected faecal samples are more likely to provide evidence of other classes of residents such as floaters as well as dispersing or nomadic lynx, none of which are likely to habituate to traps, and thus increase the measurement of genetic diversity. These animals are part of the same population–therefore measures which include these lynx would more accurately reflect the genetic diversity of the entire population.

In this study we conducted the first assessment of the spatial organisation (female philopatry, male dispersal and relatedness) and genetic variability of the northwest Anatolian *L*. *l*. *dinniki* population using nuclear microsatellite markers with the help of non-invasively and invasively sampled material. We examined the genetic variability of this potentially isolated Eurasian lynx population and evaluated it in the context of similar data for Eurasian lynx populations from central and Eastern Europe. In addition, we tested the predictions from our hypothesis and compared measures of genetic diversity obtained from different sample sources (invasive vs. non-invasive) to provide insights into the effect of different sampling methods on estimates of genetic diversity in a territorial carnivore at a small spatial scale.

## Materials and methods

### Study area

All samples were collected in an area of 396 km^2^ in the Nallıhan Mountains (40°11’- 31°21’; Fig 1), which is a mountain chain that lies in the transition zone between the dry western Black Sea (xero-euxine) and central Anatolian (Iran-Turan) floristic zones. The area does not hold any form of protection status, and is part of the state forests management system. This region is also influenced by the Mediterranean floristic zone (western Aegean), through the catchment area of the Sakarya River [31]. Vegetation and landscape have been shaped by altitude and historical human use. The lower areas (500 to 1000 m) are covered by steppe in the south, which is gradually replaced by Turkish pine (*Pinus brutia*). Above this belt, temperate coniferous forest reaches up to 1500 m and is composed of black pine (*Pinus nigra*) and junipers (*Juniperus excelsa* and *J*. *oxycedrus*) with an understory of oak-dominated scrub (*Quercus pubescens*, *Pyrus elaeagnifolia*, *Crataegus* spp., 29) with frequent forest openings. The mean annual temperature is 9.6° Celsius and the mean annual total precipitation is 543 mm [32]. The human population in this area is at a low density and restricted to several villages in the surrounding lowland and valleys. Red deer (*Cervus elaphus*) and wild boar (*Sus scrofa*) are the common large herbivores, and brown hare (*Lepus europaeus*) is the main lynx prey species here [15]. The area is home to several other large and medium-sized carnivores, at higher elevations brown bear (*Ursus arctos*) and grey wolf (*Canis lupus*) are sympatric with lynx, and at lower elevations golden jackal (*Canis aureus*), red fox (*Vulpes vulpes*) and jungle cat (*Felis chaus*) occur, which rarely occur in lynx and wolf habitat [33].

### Sample collection

In total, 183 samples were collected between November 2013 and March 2017. Swab samples taken from the outer layer of lynx faeces (N = 171; [34]) were collected by walking on active lynx trails, dirt roads and ridgelines at altitudes ranging from 1000 to 1500 meters above sea level (asl), in the Nallıhan Mountains (Fig 1). In order to reduce the chance of falsely identifying faeces from other wild carnivores and domestic dogs as lynx faeces, we used a scat detection dog [35] trained on Caucasian lynx faeces collected at Ankara Zoo. Additionally, we also applied identification criteria to correctly assign lynx faeces based on shape, segmentation (i.e. well-defined tapered segments [36]) and diameter [37]. Lynx faeces were also collected for the purpose of diet analysis [15]. Based on visual inspection, faeces varied considerably in age. We collected the samples in an area (396 km^2^ in total) that covered the territories of individually recognised male (n = 5) and female (n = 6) lynx that had previously been repeatedly identified over several years by camera trapping (S2 Table). By repeatedly searching the study area, we collected 171 faecal samples on 52 survey days (Table 1).

**Table 1:** Summary of parameters for invasively and non-invasively collected samples.

	non-invasively collected	invasively collected
effort (days*)	52	961
number of territories covered	11	10
number of territories sampled**	9	9
samples collected	171	12
samples collected per day	3.28	0.01
	11 loci / 8 loci	11 loci / 8 loci
genotyped samples	27 / 45	11 / 12
distinct genotypes	10 / 14	11 / 12
territorial lynx	7 / 8	6 / 6
kittens	1 / 2	3 / 4
dispersers and floaters***	2 / 4	2 / 2

*search days for non-invasive sampling, trapping days multiplied by active traps

**successful genotyping

***dispersers are subadult dispersing and floaters are adult non-territorial resident lynx that use much larger home ranges than territorials [30]

Capture of lynx and field work were performed in collaboration with the Wildlife Department of the Turkish Ministry of Agriculture and Forestry (WDT) under protocol and permit number 30057506-030-1867 issued by the department. Five cage traps constructed by the WDT (length: 2 m, height: 1.5 m, width: 1 m) were used for capturing lynx. Captured lynx individuals were anaesthetised and sampled by the authorised wildlife veterinarian of the WDT following national ethical legislation. No specific permit was required for anaesthesia and lynx treatment as it was conducted by the WDT. Traps were placed on lynx trails at nine trapping stations in the territories of four male and six female lynx (Table 1). We monitored the traps by GPRS camera traps (Keepguard KG860, Keeptime industrial (Asia) Co., LTD, Hong Kong, CHINA) and VHF transmitters. Each trap was visited and checked every second day. Over the course of three trapping seasons (= 961 active trap days between December and April during the years 2015–2017), we obtained “invasive” samples from 12 lynx caught in traps at five trapping stations, three in 2015, five in 2016 and four in 2017. “Invasive” samples (n = 12) were collected as small ear tissues (n = 9), a mouth swab (n = 1) and plucked hair from kittens (n = 2). For the anaesthesia of 9 lynx, 5mg/kg ketamine and 0.2mg/kg medetomidine were used. They were fitted with 185 g GPS collars (e-obs GmbH, Grünwald, Germany). One old adult male captured in 2015 and two kittens captured in 2016 were neither anesthetised nor collared because of unsuitable age and ethical concerns.

### DNA extraction and genotyping

DNA was extracted from all sample types using a commercially available forensic DNA extraction kit (GEN-IAL GmbH, Troisdorf, Germany) following the manufacturer’s instructions. As no other felid species was present in the lynx habitat (1000 m to 1500 m asl) and golden jackals and red fox were distributed at lower elevations close to human settlements due to interference competition by wolves and lynx [33], we did not apply genetic species identification. PCR cycling conditions were the same as in Bull et al. [14]. All samples were genotyped at fourteen microsatellite loci originally derived from the domestic cat (*Felis* ca*tus*) [38, 39] and the Canada lynx (*Lynx canadensis*) [40]: FCA006, FCA008, FCA082, FCA097, FCA105, FCA229, FCA441, FCA478, FCA506, FCA718, FCA1023, F115, LCA109 and LCA110. We also genotyped samples at two sexing loci: amelogenin and zinkfinger (F-AMEL and Z-Zf). One of each primer pair was labeled with a fluorescent dye (6-FAM, HEX) and loci were amplified in 5 multiplexes of 10 μL final reaction volume, applying the recommended conditions by the multiplex PCR kit manufacturer (Qiagen Multiplex PCR Kit, Qiagen, Hilden, Germany).

As genotypes determined from non-invasive samples may be incomplete or suffer from errors (e.g. allele dropout, false alleles), we applied a maximum likelihood approach [41] to ensure that genotypes were reliably identified. We genotyped each faecal sample in duplicates and only retained samples that had consistent allele calls in both amplifications. If a mismatch was observed, a new DNA extraction of the same sample was carried out and the procedure was repeated (parallel genotyping). Thus, every sample was genotyped two or four times. If there was no further material left for a second extraction round, or if the second round of duplicate genotyping also showed mismatches, the respective sample was discarded. We retained genotypes that included consistent amplifications at 12 or more loci (but see below).

Given the large number of samples taken in the field and the size of the area surveyed, multiple sampling of some individuals was expected. Applying the option ‘alleleMismatch = 2’ of the software *Allelematch* version 2.5 [42], we compared genotypes and assigned multiple samples to the same genotype (i.e. individual). This included genotypes that did not match because of size shift in one allele (N = 3) and/or missing data (N = 3). To quantify the discriminatory ability of our loci, we estimated the cumulative values of the unbiased probability of identity (P_*IDunb*_) and probability of identity given siblings (P_*IDsib*_) using the software GIMLET version 1.3.3 [43].

### Genetic analyses

The probability for the presence of null alleles at the fourteen microsatellite loci was estimated using the software MICROCHECKER version 2.2.3 [44]. Potential deviations from Hardy-Weinberg equilibrium (HWE) and presence of linkage disequilibrium (LD) were both tested using GENEPOP version 3.4 [45]. We used FSTAT version 2.9.3.2 [46] to estimate the inbreeding coefficient (*F*_IS_) and expected (*H*_E_) and observed heterozygosities (*H*_O_). Allelic richness (*A*_R_) and Kosman and Leonard’s measure of genetic dissimilarity versus geographic distance [47] were determined using the R package *PopGenReport* version 2.1 [48]. *A*_R_ was estimated using rarefaction of eight genotypes per population (except for Slovakian lynx, N = 6). Kosman and Leonard’s measure of genetic dissimilarity was applied to visualize the spatial organisation and pairwise relatedness among territorial lynx (i.e. excluding the kittens and other individuals without territories) in the study area. This required a spatial coordinate to represent the individuals included in the analysis. The procedure to allocate the appropriate spatial coordinate is explained below. Additionally, we conducted a spatial autocorrelation analysis using GenAlEx version 6.502 [49, 50]. To obtain equal numbers of comparisons per distance class, we used the “even sample classes” option. Intra-population pairwise relatedness (M_xy_ [51]) values were estimated using the R package *Demerelate* version 0.9–3 [52].

We also used our microsatellite data and reanalysed them in combination with the data of Bull et al. [14] using the ten microsatellite loci shared in these studies (FCA006, FCA008, FCA082, FCA097, FCA105, FCA229, FCA506, FCA718, FCA1023, and LCA110). This enabled us to compare northwest Anatolian lynx and autochthonous and reintroduced Eurasian lynx populations in central and Eastern Europe in terms of genetic diversity and intra-population relatedness.

### DNA sampling method and diversity measures

In order to evaluate whether the two sampling methods (“invasive” vs. “non-invasive”) affected estimates of population genetic diversity, we needed more samples with complete genotypes. Therefore, we removed three microsatellite loci that had generated missing data for numerous samples. The eight loci for which additional samples had a complete genotype were FCA008, FCA082, FCA097, FCA105, FCA229, FCA1023, LCA109 and LCA110. For this aforementioned comparison, we considered the mean number of alleles ($\bar{N}$) and the expected heterozygosity (*H*_E_) as measures for genetic variability, estimated using the function ‘subsample.gen’ of the R package *Resamplediversity* version 1.0 [53]. This function allowed us to consider various sizes of subsamples of our genotypes (separately for the invasive and non-invasive samples, as well as for the combined sample set), with sizes ranging from 2 to 22, with 100 iterations per number of genotypes. In this manner, we tracked how an increase in sample size changed the estimates of $\bar{N}$ and *H*_E_. We then used Tukey’s test to examine whether $\bar{N}$ and *H*_E_ values differed significantly between sample types. The test was conducted for the range of genotypes from 2 to 12, the latter being the maximum number of genotypes among invasive samples. Bonferroni’s inequality method [54, 55] was used to adjust the significance threshold by the number of comparisons, resulting in an adjusted significance threshold of α = 0.0015.

### Lynx population monitoring

Along with ‘non-invasive’ and ‘invasive’ genetic sampling, the lynx population had also been monitored by camera traps at 54 different stations since autumn 2009 (S2 Table) and by recording the movements of nine individual lynxes had been tracked using GPS transmitters since 2015. We matched lynx genotypes from non-invasively collected samples to individuals (morphology) if the sample from a particular individual had been collected from a camera-trapping station with evidence of a picture having been taken during defecation, or when the faeces were found in very close proximity (maximum distance of 5 m) of a camera trap station in the following two days after the picture had been taken. Faeces (and their corresponding genotypes) were categorized as belonging to a kitten, when the faeces diameter was smaller than the diameter of adult lynx faeces [37], there was no picture of the defecating individual and the faeces was found in the territory of a female lynx to which the pairwise relatedness of the genotype was higher than 50%.

One spatial coordinate per individual was used in the analysis of the spatial organisation of territorial lynx in our study area for the genetic dissimilarity vs. distance analysis. If the territorial resident individual was collared (n = 5), we used the home range centroid estimated from GPS data as the spatial coordinate. For the remaining territorial residents (n = 5), we used the spatial coordinates of the centroid of the minimum convex polygon established from locations where faecal samples had been collected with this genotype and the locations of camera traps where pictures of that particular individual had been taken.

## Results

Except for the monomorphic locus FCA478, which was excluded from further analyses, all other microsatellite loci were polymorphic, with the number of alleles (*N*_A_) ranging from three to seven (Table 2). No combination of microsatellite loci was in linkage disequilibrium (LD) but two loci (F115, FCA441) had a significant probability for the presence of null alleles. These two loci also showed signs of inbreeding (as measured by *F*_IS_) and deviated significantly from Hardy-Weinberg equilibrium (HWE, Table 2). Therefore, these two loci were omitted in subsequent analyses.

**Table 2:** Summary of genotyping results at 14 microsatellite loci for north-western Anatolian lynx.

Locus	*N*_A_	*H*_E_	*H*_O_	*p*_HWE_	*F*_IS_	*f*_*null*_ [56]
FCA006	3	0.68	0.76	n.s.	-0.130	-0.07
FCA008	3	0.62	0.87	n.s.	-0.405	-0.17
FCA082	7	0.80	0.82	n.s.	-0.032	-0.03
FCA097	5	0.64	0.72	n.s.	-0.122	-0.06
FCA105	4	0.74	0.61	n.s.	0.176	0.08
FCA229	3	0.52	0.61	n.s.	-0.176	-0.09
FCA441†	7	0.83	0.35	<0.01	0.571*	0.37*
FCA478†	1	0	0	n.a.	n.a.	0
FCA506	5	0.44	0.35	n.s.	0.200	0.09
FCA718	5	0.72	0.87	n.s.	-0.201	-0.1
FCA1023	5	0.76	0.65	n.s.	0.150	0.06
F115†	3	0.62	0.08	<0.01	0.866*	0.74*
LCA109	3	0.67	0.67	n.s.	0.012	-0.01
LCA110	5	0.59	0.61	n.s.	-0.078	-0.05
Average across 11 loci	4.45	0.65	0.69		-0.050	

* p < 0.05

† microsatellite loci removed from subsequent analyses

Number of alleles (*N*_A_), expected (*H*_E_) and observed (*H*_O_) heterozygosity, probability of deviation from Hardy-Weinberg equilibrium (*p*_HWE_), inbreeding coefficient (*F*_IS_), estimated frequency of null alleles (*f*_*null*_).

### Relatedness and spatial organization

**Fig 2:**
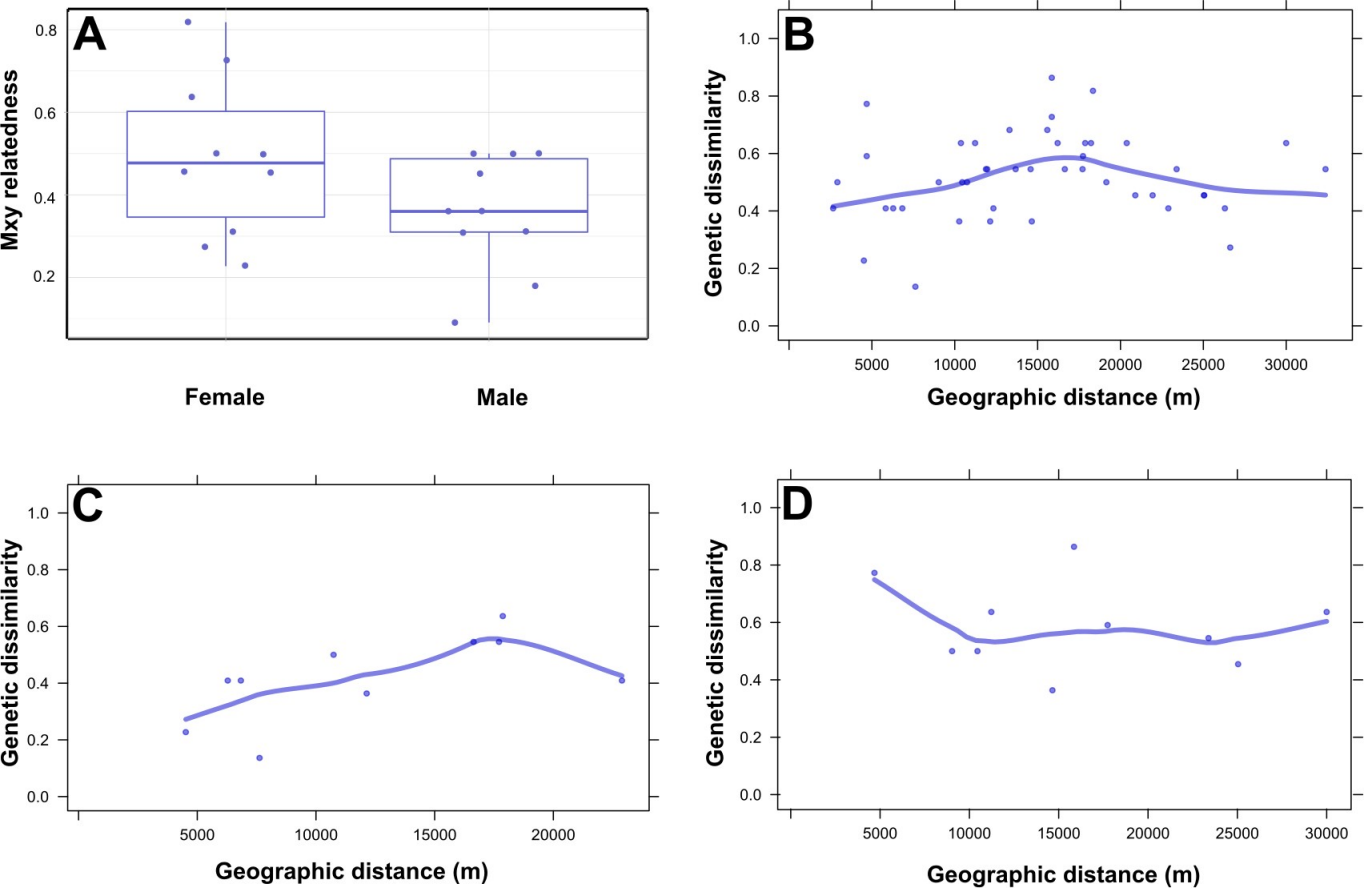
Relatedness and spatial organization of territorial lynx in northwestern Anatolia. *M*_*xy*_ relatedness values (A) of female and male territorial lynx in northwestern Anatolia. Plots of genetic dissimilarity (Kosman and Leonard, 2005; n_loci_ = 11) versus geographic distance, for (B) all territorial lynx (n_genotypes_ = 10), (C) for territorial females (n_genotypes_ = 5) and (D) territorial resident males (n_genotypes_ = 5).

The analysis of pairwise relatedness of lynx in the study area revealed that territorial females had a wide range of relatedness within the study area, including three female-female pairs that were highly related (consisting of two different groups of mother-daughter pairs; Fig 2A). Territorial male-male pairs generally showed lower relatedness, including two highly unrelated pairs (Fig 2A). The mean genetic dissimilarity among territorial lynx (n = 10) varied by distance. We found a moderate increase in dissimilarity over shorter distances of up to 17 km, after which it declined (Fig 2B). When the sexes were considered separately, neighbouring territorial female lynx displayed high similarity, and dissimilarity peaked at 17 km (Fig 2C), corresponding to a distance of three female territories in the study area. Neighbouring males showed high dissimilarity and none of the territorial males were close relatives (Fig 2D). The results of the spatial autocorrelation analysis were broadly consistent with these finding. There was a significant positive correlation (*r* ≥ 0.12) at distance classes up to 7 km (*P* ≤ 0.05) and a significant negative correlation (*r* ≤ -0.11) at distance classes of 13 km and 16 km (*P* ≤ 0.05) (S1 Fig). In order to conduct the spatial autocorrelation analysis with a sufficient number of samples we had to include all genotypes (including kittens, dispersers and floaters); sample deficiency was the reason why the analysis could not be separately performed for females and males.

### Genotyping success and genetic diversity measures

Amplification success of the 11 loci across all samples was 66%. Among the 171 ‘non-invasive’ samples, 27 (15.8%) were successfully genotyped twice at nine to eleven microsatellite loci (i.e. with consistent allele calls). Among the ‘invasive’ samples, all 9 tissue samples, the mouth swab and one out of two plucked hair samples generated data for the same number of loci (91.7%). Among the 27 non-invasive and 11 invasive samples (n = 38), we detected 18 unique lynx genotypes (7 females and 11 males), 10 from non-invasive and 11 from invasive samples; three genotypes were detected using both sources. The cumulative estimates of probability of identity were: *P*_IDunb_ = 1.02 × 10^−9^ and *P*_IDsib_ = 3.12 × 10^−4^. The mean *A*_R_ was 3.96, mean *H*_E_ and *H*_O_ were 0.65 and 0.69, respectively, and mean *F*_IS_ was -0.055.

**Fig 3:**
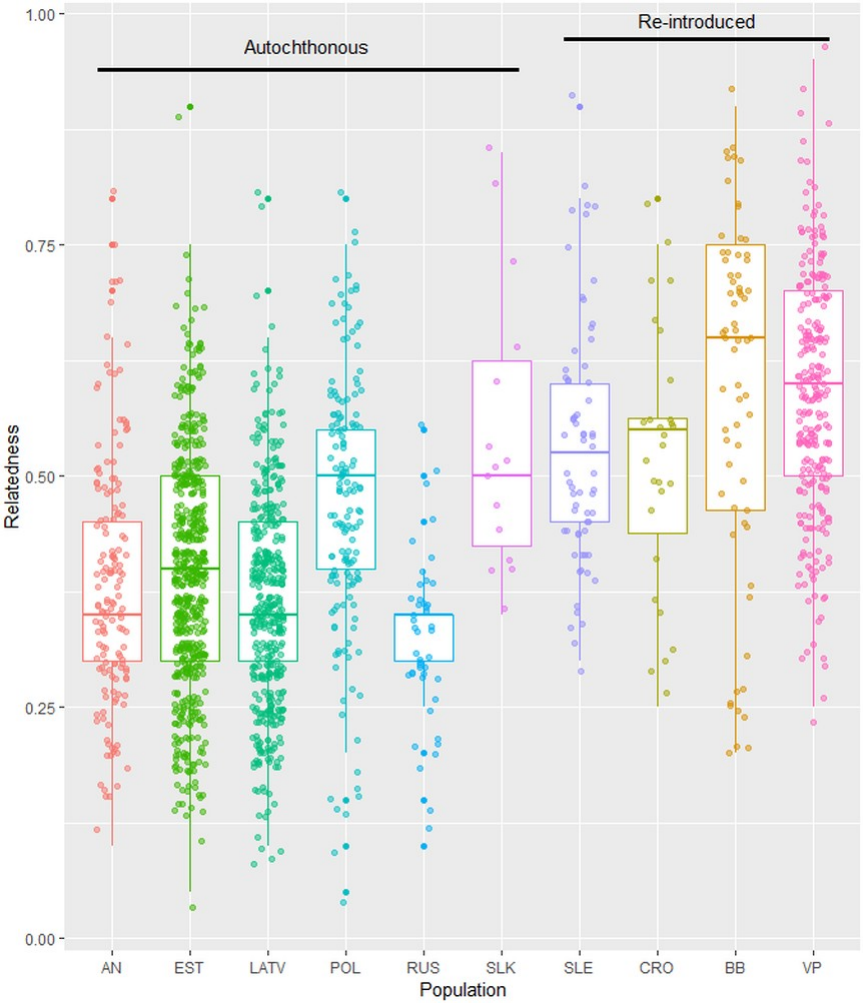
Relatedness (*M*_*xy*_) among individuals in Eurasian lynx populations, including northwestern Anatolia and autochthonous and re-introduced lynx populations of central and eastern Europe (based on reanalysis of 10 shared microsatellite loci [14]).

When one locus was removed from the dataset and measures of genetic diversity were re-estimated from the 10 microsatellite loci matching the ones previously employed by Bull et al. [14], the mean diversity measures of lynx in Anatolia were not affected (Tables 1 and 2). Re-analysis of our dataset and data from Bull et al. [14] showed that genotypes from Anatolia (n_genotypes_ = 18, n_loci_ = 10) had the second highest *A*_R_ and *H*_O_ values after the lynx population from Russia and the second lowest *F*_IS_ value after the lynx population from Slovakia (Table 3). Among the autochthonous *L*. *lynx* populations, Anatolian, Latvian and Russian lynx had the lowest mean relatedness, followed by Estonian lynx (Fig 3). Two autochthonous lynx populations (Poland and Slovakia) displayed a higher relatedness, with values closer to that of reintroduced European lynx populations. Among reintroduced European lynx populations, the lynx populations from the Bohemia-Bavarian and Vosges-Palatinian areas had the highest relatedness values (Fig 3). Using the same reduced dataset (n_genotypes_ = 18, n_loci_ = 10), an analysis of pairwise relatedness (*M*_*xy*_) revealed 14 full-sibling/parent-offspring pairs (*M*_*xy*_ threshold = 0.59), 37 half-sibling pairs (*M*_*xy*_ threshold = 0.43), and 102 pairs of unrelated individuals for lynx in northwest Anatolia.

**Table 3:** Comparison between the northwestern Anatolian lynx population and other autochthonous and reintroduced European lynx populations [14], based on reanalysis of 10 shared microsatellite loci.

Population	*N*	*A*_R_	*H*_E_	*H*_O_	*F*_IS_
NW Anatolia	18	3.62	0.65	0.69	-0.055
*Other autochthonous populations*					
Estonia	34	3.57	0.67	0.67	0.004
Latvia	29	3.52	0.70	0.66	0.064
Poland	18	3.17	0.60	0.59	0.014
Russia	10	3.74	0.73	0.71	0.033
Slovakia	6	2.90	0.57	0.63	-0.121
*Reintroduced populations*					
Bohemia-Bavaria	12	2.61	0.46	0.44	0.044
Vosges-Palatinian	23	2.57	0.49	0.47	0.042
Croatia	8	2.91	0.53	0.46	0.132
Slovenia	12	2.81	0.54	0.51	0.059

Numbers of genotypes (*N*), allelic richness (*A*_R_), observed (*H*_O_) and expected (*H*_E_) heterozygosity, inbreeding coefficient (*F*_IS_).

### Sampling method and diversity measures

Over 52 survey days we collected 171 faecal samples (mean: 3.3 samples/ day) with the help of a scat detection dog (Table 1). In 961 trapping days we trapped and sampled 12 lynx (mean: 0.01 samples or animals/ day), visiting each trap every other day and renewing lures (i.e. lynx urine). We obtained a similar number of genotypes from both sampling approaches (Table 1), but needed a 19-fold higher effort in the ‘invasive’ sampling approach.

A reduction of the number of loci to eight microsatellites (see Methods) increased the number of unique genotypes to 22 among 57 samples. This larger dataset included one additional genotype that was detected using the two sample types and increased the number of overlapping genotypes to four. We identified 14 unique genotypes among 45 non-invasively collected samples and 12 unique genotypes from the 12 invasively collected samples (Table 1). Cumulative estimates of probability of identity using the eight microsatellite loci were *P*_IDunb_ = 3.79 ×10^−7^ and *P*_IDsib_ = 2.67 × 10^−3^.

**Fig 4:**
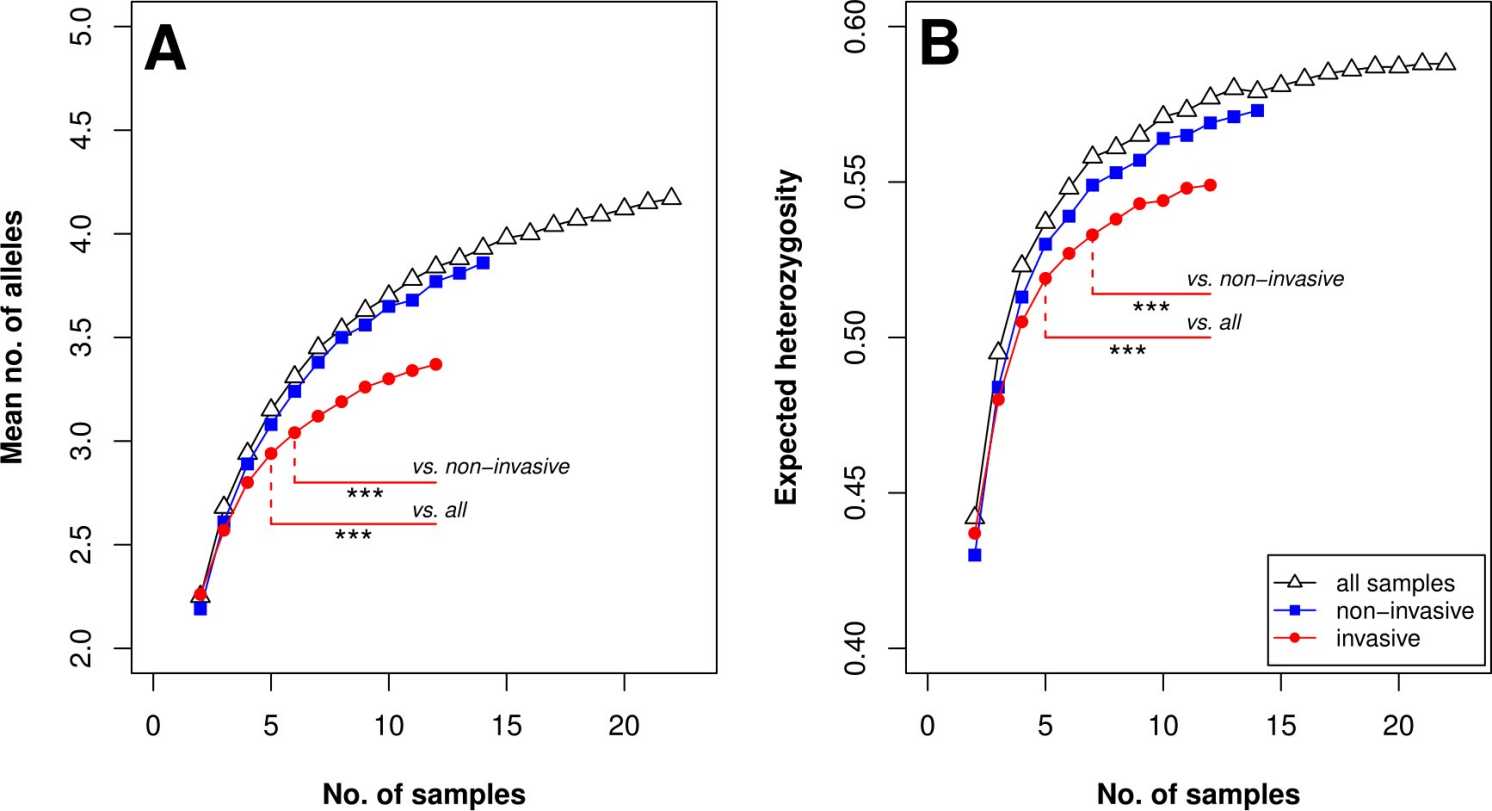
Accumulation rates of diversity measures with increasing sample numbers per sample type. A) Mean numbers of alleles ($\bar{N}$), and B) expected heterozygosity (*H*_E_) values for genotypes sampled non-invasively (N = 14), invasively (N = 12) and for all genotypes (N = 22), using 8 microsatellite loci.

When we applied the subsampling analysis, the curves depicting the accumulation of mean number of alleles ($\bar{N}$) and expected heterozygosity (*H*_E_) differed between the two sampling methods (Fig 4). For both measures, genotypes from invasively collected samples were significantly less diverse than those from non-invasively collected samples (after subsamples of 6 or 7 genotypes; Tukey’s test, p$\bar{N}$ < 0.0015, p*H*_E_ < 0.0015) or if all samples were combined (after subsamples of 5 genotypes; Tukey’s test, p$\bar{N}$ < 0.0015, p*H*_E_ < 0.0015). The diversity observed among genotypes from non-invasively collected samples did not significantly differ from the diversity measured among all genotypes (for all subsample comparisons; Tukey’s test, p$\bar{N}$ > 0.0015, p*H*_E_ > 0.0015).

### Lynx population monitoring

Along with camera trapping and GPS collaring of lynx, genotyping enabled us to monitor 18 lynx individuals for periods from 6 months to 8 years (S2 Table). Nine lynx individuals were monitored by means of camera trapping, genetic monitoring and GPS tracking, and the remaining nine lynx by camera trapping and genetic monitoring but without GPS tracking. We matched seven of the unique lynx genotypes with known lynx morphologies from lynx visits and defecation in front of or very close to camera trap stations. Except for one subadult (monitored for 6 months) these lynx were territorial residents monitored over several years. Three of the individuals caught in the cage traps were genetically sampled (hair from two kittens and tissue from an old male) but not GPS tracked. We monitored the 18 lynx over a mean of 3.1 ± 2.2 years using the combination of camera trapping, genotyping (non-invasive and invasive sample sources) and GPS tracking. The remaining four individuals were only identified by faecal genotypes. Unfortunately, these lynx could not be matched to camera trap pictures because defecation had not occurred in proximity to a camera trap.

## Discussion

In this study, we report the first population genetic diversity measures for Caucasian lynx *L*. *l*. *dinniki*, focusing on the potentially isolated northwestern Anatolian population. We consider the diversity of *L*. *l*. *dinniki* in the context of previously published data for autochthonous and reintroduced Eurasian lynx populations in Europe (subspecies *L*. *l*. *lynx* and *L*. *l*. *carpathicus*), and consider the consequences of using invasive sampling versus non-invasive sampling for measures of genetic diversity of this territorial felid.

### Genotyping

Genotyping is a valuable tool for assessing population genetic status and viability of endangered or data deficient animal populations [8, 57]. Planned and applied conservation activities such as captive breeding or re-introduction projects of endangered species use population genetics as a tool to measure genetic diversity in wild populations (e.g. *Lynx pardinus* [58]). Most preliminary conservation activities and conservation genetics studies of wild populations start in small survey areas, or are locally restricted because of restricted distribution of the target populations (e.g. *L*. *pardinus* [58]; *L*.*l*. *balcanicus* [59]; *Panthera pardus orientalis* [60]; *Panthera pardus melas* [61]). Small survey areas might in turn result in lower genetic diversity estimates.

Two sampling strategies are common: ‘invasive’ and ‘non-invasive’. As our study is the first one on the *L*. *l*. *dinniki* population in northwestern Turkey, and because we were interested in generating baseline information on population genetics, we used both approaches. The ‘invasive’ sampling strategy was applied to ensure reliability of genotyping results, the ‘non-invasive’ sampling strategy was applied because it would increase the number of samples available for the study. The method was even improved by employing a scat detection dog. Having samples from both sampling approaches also provided an opportunity for a comparison of the results obtained in both approaches. Future surveys of the northwestern population and other populations of Caucasian lynx in Turkey, Caucasus and Iran could then potentially rely on data from ‘non-invasive’ sampling.

As described in other studies, genotyping success in faecal samples can be a relatively low in relation to sampling effort and this can vary among species [62, 63]. In our study we attribute the low amplification success of non-invasive samples to the unknown and highly varying time lengths for which the faeces were exposed to environmental influences. We did not restrict ourselves to collecting fresh faecal samples only, because older faeces would also be useful for the purpose of diet analysis [15]. Long exposure time may not just influence genotyping success per se, but may also cause genotyping errors that need to be accounted for and which may also vary across species [5, 64–66]. Our genotyping results suggest that studies focused on genetic analyses should emphasize the collection of samples from freshly defecated faeces.

### Spatial organisation

The spatial genetic analysis of the territorial members of northwestern Anatolian lynx population (combined sampling sources) revealed a unimodal genetic dissimilarity pattern, with a peak at 17 km (line in Fig 2B). Pairwise comparisons revealed that territorial females were most similar to each other at distances of up to 8 km, indicating that mothers and their daughters held neighbouring territories. The most dissimilar female pairs were observed at distances of 17 km (Fig 2C). No closely related males occupied neighbouring territories, indicating that male offspring of territorial males establish territories at larger distances from the territory they were born in. In our study area, the mean distance (MD) between the territory centres of territorial males was 12.1 km ± 3.1 km and 7.1 km ± 2.5 km for territorial female lynx (DM unpublished data). The results of the spatial autocorrelation analysis among lynx in our study area is consistent with this finding, indicating the highest negative correlations (i.e. most dissimilar genotypes) at distance classes of 13 km and 16 km (S1 Fig). Therefore, to overcome the negative influence of sampling at small spatial scales (i.e., clumped sampling) on genetic diversity, sampling of female lynx neighbouring territories should be avoided as these females will very likely be closely related. A sampling design that places live traps at every second female territory would most probably capture a higher genetic diversity, while reducing the relatedness among genotypes at the same time. In our study area, this would correspond to a minimum distance of 15 km between traps and would need to be specified for other Eurasian lynx or philopatric carnivore populations, and depend on their respective densities.

### Impact of spatial organisation on measured genetic diversity

We observed substantial differences between measures of genetic diversity of a single lynx population derived from two sampling approaches (Fig 3), for which we identified four reasons: First, systematic ‘non-invasive’ sampling (in our study with a scat-detection dog) is more likely to sample the population evenly, both due to the larger number of samples to be collected and the much higher number of locations covered. Second, the chance of non-invasively sampling a resident floater or dispersing individual is much higher than the chance of cage-trapping a member of this segment of the population. Whereas resident floaters or dispersing animals remain in a particular area only for a few days, it is likely that they will leave traces such as one to two faeces/day [67] during a visit, which can be detected during non-invasive sample collection for some time after these individuals left the area again. Because box or cage traps stay in their locations for long periods of time, (often over months or even years; including inactive non-trapping periods), territorial lynx become accustomed to them. The chance of trapping territorial lynx and their kittens is therefore higher (S2 Fig and S1 Video) than the chance of trapping visiting lynx individuals such as resident floaters or dispersing individuals, which are less likely to be habituated to the traps. Third, to increase the chance of trapping territorial resident lynx, trap stations are placed in locations that are frequently visited by residents, such as lynx marking sites or on frequently used trails. These locations are often determined by prior camera trapping and are generally either in the core areas of lynx territories or are located in the overlapping ranges of several adult lynx. Fourth, female philopatry can further enhance the effect of sampling protocol on diversity measures if samples are collected in neighbouring female territories, thereby increasing the chance of collecting samples from related territorial individuals (e.g., mothers and their daughters).

If we had only used invasive samples for genetic monitoring, as was done for many preliminary conservation projects for endangered species [60, 68, 69], we would have underestimated the genetic variability in our study population. Therefore, our results emphasize the importance and usefulness of non-invasive sampling for conservation genetics studies of endangered and data deficient territorial carnivore populations, particularly at small spatial scales.

### Genetic diversity

#### Within Anatolia

Considering its substantial diversity (*H*_E_ = 0.65, *H*_O_ = 0.69) and lack of inbreeding (*F*_IS_ = -0.05), the northwestern Anatolian lynx population currently does not appear to require any management to bolster its genetic diversity. In order to conserve its current genetic diversity, we highly recommend identification and maintenance of primary lynx habitats and corridors in northwest Anatolia. As there is no other study on Anatolian lynx we could compare our findings with, our data provide a ‘genetic baseline’ of a seemingly healthy lynx population, available to future studies to measure anthropogenic and other impacts on this population (e.g., along a time line). Similar work is also needed for the other two Turkish populations of *L*. *l*. *dinniki* in order to determine whether the three big populations in Anatolia (Fig 1) are currently connected by gene flow.

#### Comparison with other populations

Compared with other autochthonous and re-introduced lynx populations in central and Eastern Europe (Table 3 and Fig 2), only the (presumably much larger) Russian lynx population had higher values for its genetic diversity indices (e.g. *A*_R_ and *H*_O_) than the north-western Anatolian population. We observed a low mean relatedness in the northwestern Anatolian population, similar to that observed for autochthonous populations sampled over much larger geographic areas (e.g. Latvia, Russia; Fig 3).

### Lynx population monitoring

**Fig 5:**
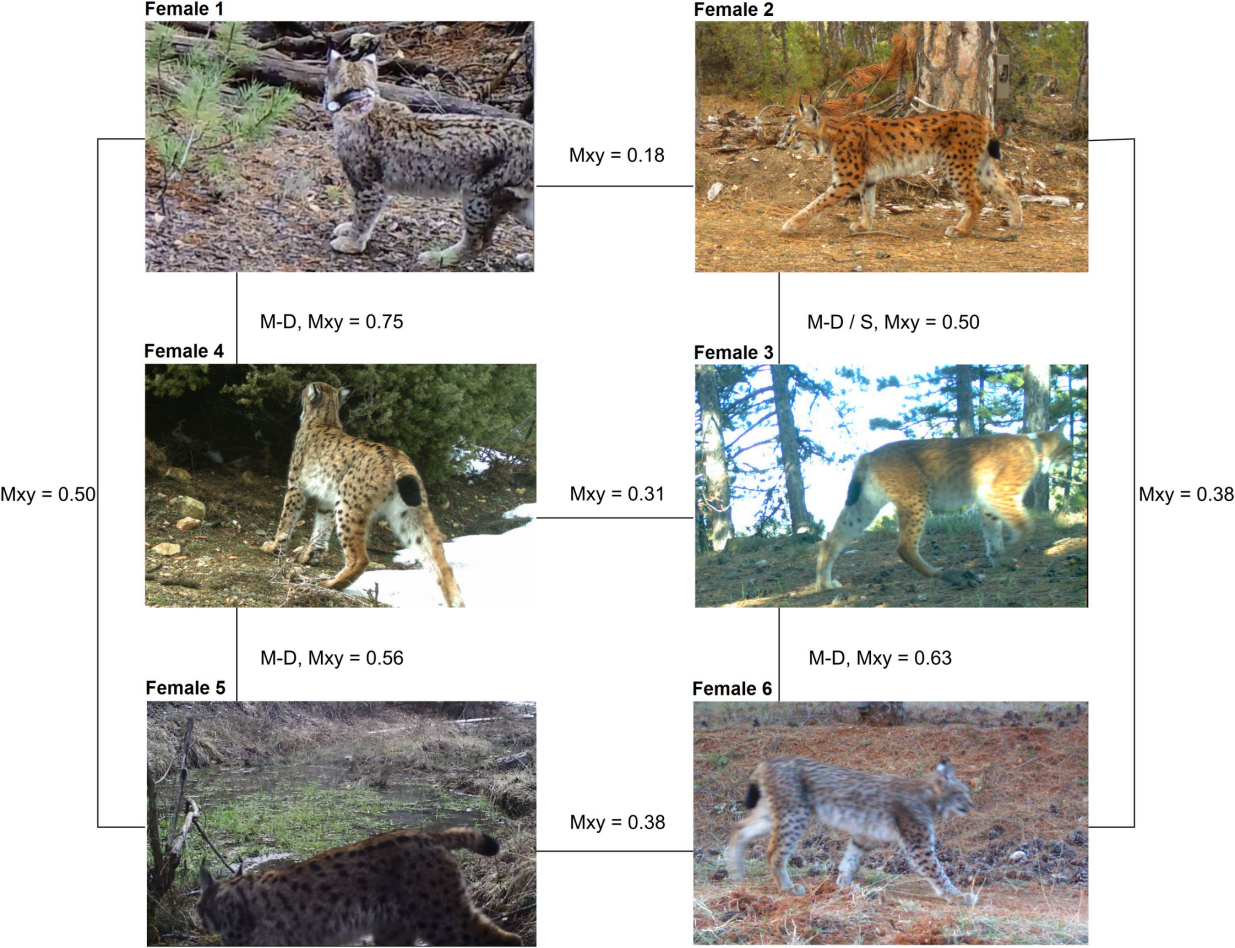
Coat patterns and *M*_*xy*_ relatedness values of territorial (No 1–5) and subadult (No 6) female lynx in NW Anatolia. Mother–Daughter (M-D), Mother–Daughter or Siblings (M-D / S).

Non-invasive genetic monitoring of carnivore populations is being increasingly used in wildlife studies. When combined with invasive sampling and camera trapping, this technique can provide valuable information on space use, marking behaviour and survival, and reveal interactions between individuals or groups [70]. By genotyping and re-sampling lynx individuals in this study, we obtained data on population dynamics, genetic relatedness, space use and other issues such as marking behaviour and spatial interactions [17] of a Caucasian lynx population for the first time. Genotyping revealed some dynamics between neighbouring territorial individuals such as male lynx intruding into territories of neighbours during mating time [17]. Although this population had been monitored since 2009 using camera traps, the relatedness among territorial lynx was still unclear but could be solved within our study. Besides revealing female philopatry genotyping also highlighted that shared pelage patterns (light background colour/ small dots) and thus has been assumed to be relatives, were actually unrelated, whereas others with very different pelage patterns (light background colour/ small dots vs. dark background colour/ big spots) turned out to be either a mother-offspring pair or a pair of full siblings (Fig 5). As in other species too [71, 72], these phenotypes appear to have a complex inheritance in lynx (e.g. dominance, pleiotropy), and cannot be used to infer relatedness.

Even if it is not combined with ‘invasive’ sampling and GPS tracking, in long-term studies, ‘non-invasive’ sampling along with camera trapping will serve as an important tool to monitor populations of individually recognizable animals [73]. If territorial individuals can be identified both phenotypically (e.g.by camera traps) and genotypically (via the genotyping of faeces), then linking this information will allow obtaining a much more comprehensive picture of behavioural and reproductive dynamics of the population in focus. The employment of a wildlife scat detection dog will even help to increase the success rate of such an approach.

## Conclusions

Caucasian lynx (*L*.*l*. *dinniki*) in northwestern Anatolia displayed high genetic diversity. Assessment of other Caucasian lynx populations in Anatolia and elsewhere is required to evaluate the conservation status of this subspecies. Our results show that sampling approach, territoriality and female philopatry can influence measures of genetic diversity, which may be relevant to conservation management decisions. ‘Non-invasive’ faecal sampling reduces the impact of female philopatry and territoriality on diversity measures and provides information on other important aspects of the biology and ecology of the species, which in turn can help to inform conservation management decisions.

## Supporting information

S1 Table Eighteen lynx genotyped at fourteen autosomal and two sexing loci (F-Amel and Z-Zf). Data used to estimate genetic diversity measures and pairwise-relatedness among genotypes of northwestern Anatolian lynx population (Tables 2 and 3 and Figs 2, 4 and 5, and S1). Orange color indicates the three microsatellite loci that were removed, and grey the two sexing loci.
(XLSX)

S2 Table Lynx individuals monitored in between 2009 and 2017. Camera trapping (CT), Genotyping (G), GPS telemetry (T). Bold values indicate total amount of genotypes obtained using 11 and 8 microsatellite loci.
(XLSX)

S1 Fig Results of spatial autocorrelation analysis for all lynx individuals genotyped at 11 microsatellite loci. (PDF)

S2 Fig Female 1 trapped with 11 months old male kitten. Father of this male kitten was also captured in the same trap at another occasion.
(PNG)

S1 Video Female 4 (daughter of female 1) is checking an inactive trap with her two kittens. One of the kittens in the video is Female 5, which was captured and collared next trapping season (14 months later) in the same trap.
(AVI)
