# Supplementary material for: Non-invasive faecal sampling reveals spatial organization and improves measures of genetic diversity for the conservation assessment of territorial species: Caucasian lynx as a case species
Source: PLoS One. 2019 May 10;14(5):e0216549. doi: 10.1371/journal.pone.0216549 (PMC6510455; doi:10.1371/journal.pone.0216549)
Supplement: S1 Fig — (PDF) [file pone.0216549.s003.pdf]

S1 Fig. Results of spatial autocorrelation analysis for all lynx individuals genotyped at 11 microsatellite loci. Bold indicates significant correlation (r) and probability (p).

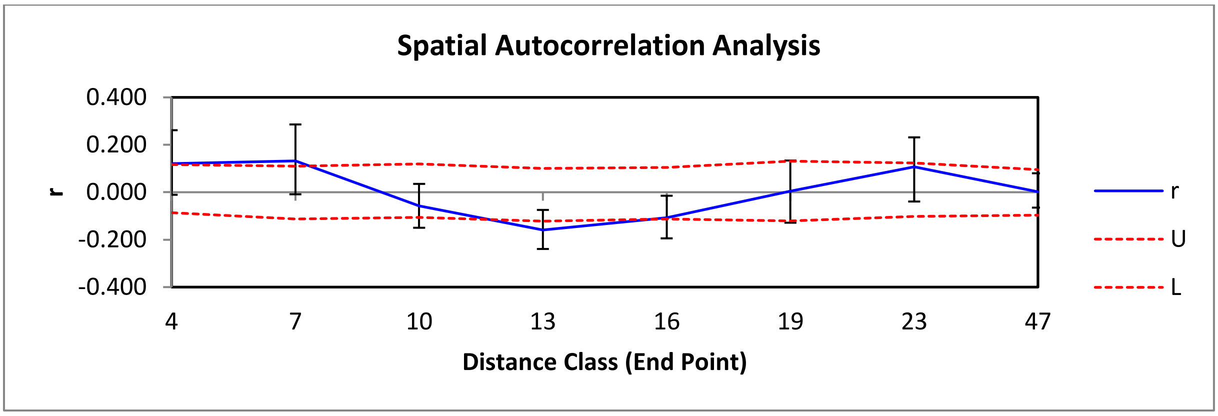

**Genetic Distance**	GD all
**Geographic Distance**	GGD all
**Data Title**	All genotypes
**No. Samples**	18
**Even Sample Sizes**	
**No. Dist. Classes**	8
**No. Permutations**	999
**No. Bootstraps**	1000

**n**	22	19	19	19	23	15	15	16
**Distance Class (End Point)**	4	7	10	13	16	19	23	47
**r**	**0.121**	**0.132**	-0.057	**-0.159**	**-0.108**	0.004	**0.107**	0.002
**U**	0.116	0.109	0.119	0.100	0.105	0.131	0.123	0.095
**L**	-0.086	-0.113	-0.106	-0.122	-0.113	-0.121	-0.103	-0.096
**P(r-rand >= r-data)**	**0.019**	**0.010**	0.853	0.993	0.973	0.456	**0.044**	0.497
**P(r-rand <= r-data)**	0.982	0.991	0.148	**0.008**	**0.028**	0.545	0.957	0.504
**Correction**	0.059	0.059	0.059	0.059	0.059	0.059	0.059	0.059
**r uc**	0.062	0.073	-0.116	-0.218	-0.166	-0.055	0.048	-0.057
**U uc**	0.058	0.051	0.060	0.042	0.046	0.072	0.064	0.037
**L uc**	-0.145	-0.172	-0.165	-0.181	-0.172	-0.180	-0.161	-0.155
**Mean Bootstrap r**	0.120	0.131	-0.055	-0.159	-0.108	0.005	0.106	0.006
**Ur error**	0.141	0.154	0.092	0.085	0.093	0.129	0.124	0.078
**Lr error**	0.132	0.141	0.093	0.080	0.087	0.133	0.146	0.067
**Ur**	0.262	0.286	0.035	-0.075	-0.015	0.134	0.231	0.080
**Lr**	-0.011	-0.009	-0.150	-0.239	-0.194	-0.128	-0.039	-0.065
**Intercept**	9.095							
