# Supplementary figures and images for: Non-invasive faecal sampling reveals spatial organization and improves measures of genetic diversity for the conservation assessment of territorial species: Caucasian lynx as a case species
Source: PLoS One. 2019 May 10;14(5):e0216549. doi: 10.1371/journal.pone.0216549 (PMC6510455; doi:10.1371/journal.pone.0216549)

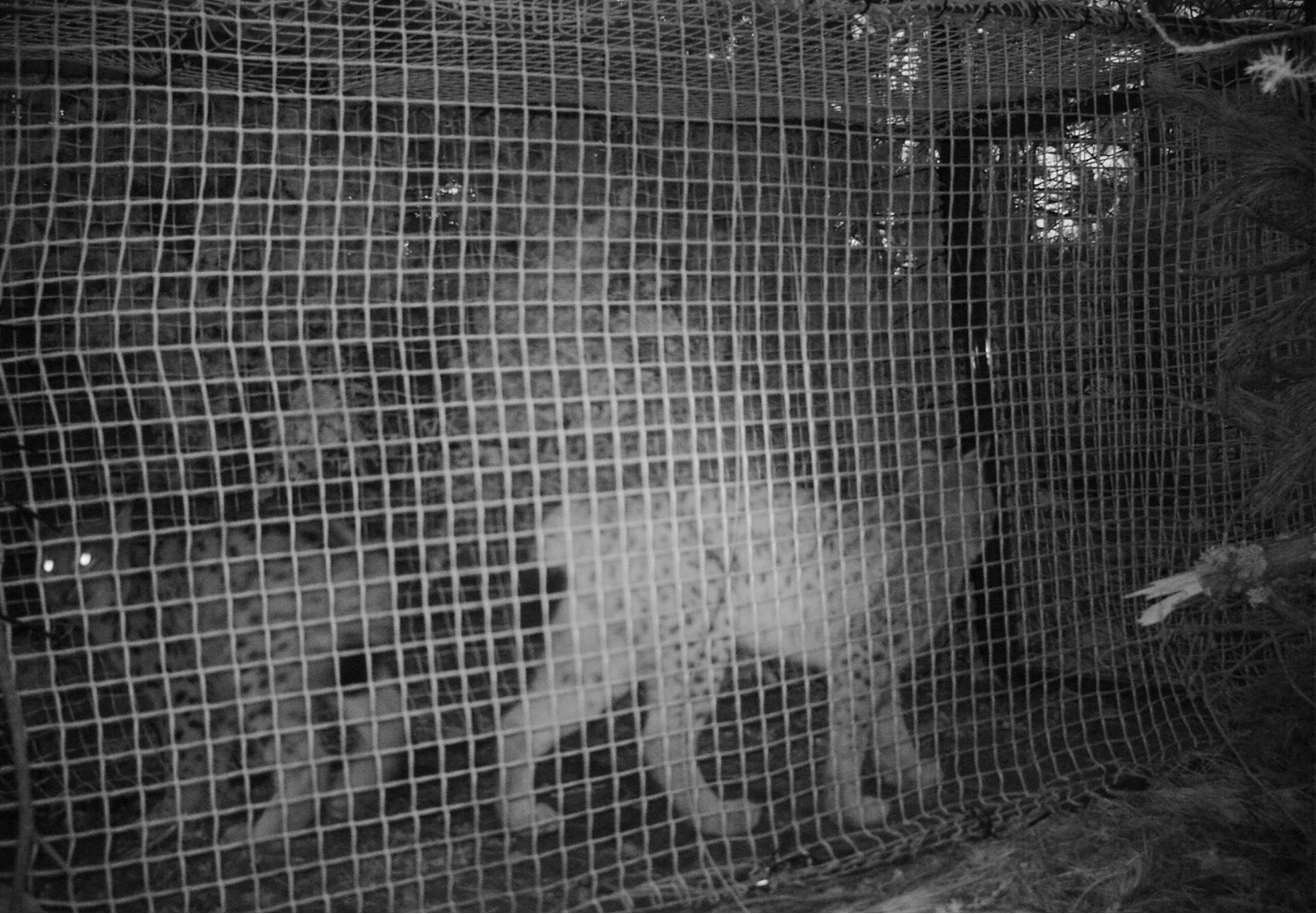

Supplement: S2 Fig — Father of this male kitten was also captured in the same trap at another occasion. (PNG) [file pone.0216549.s004.png]
